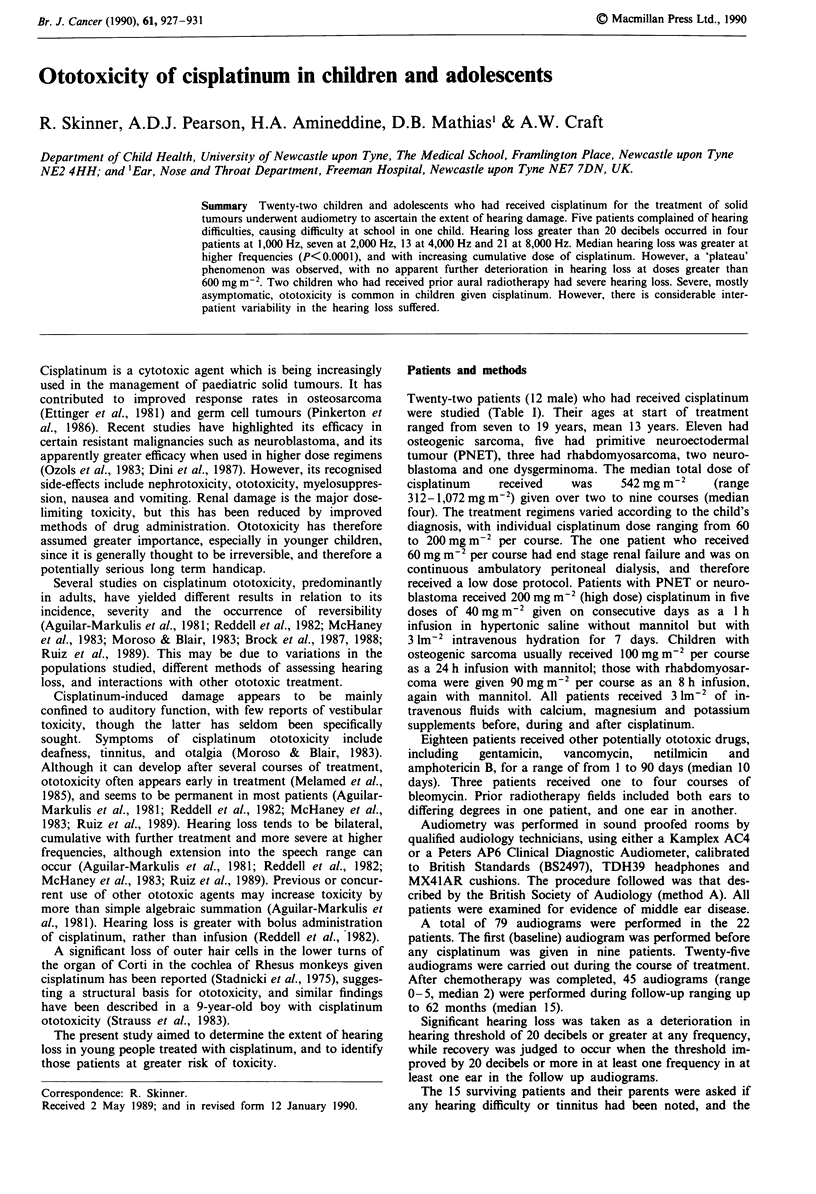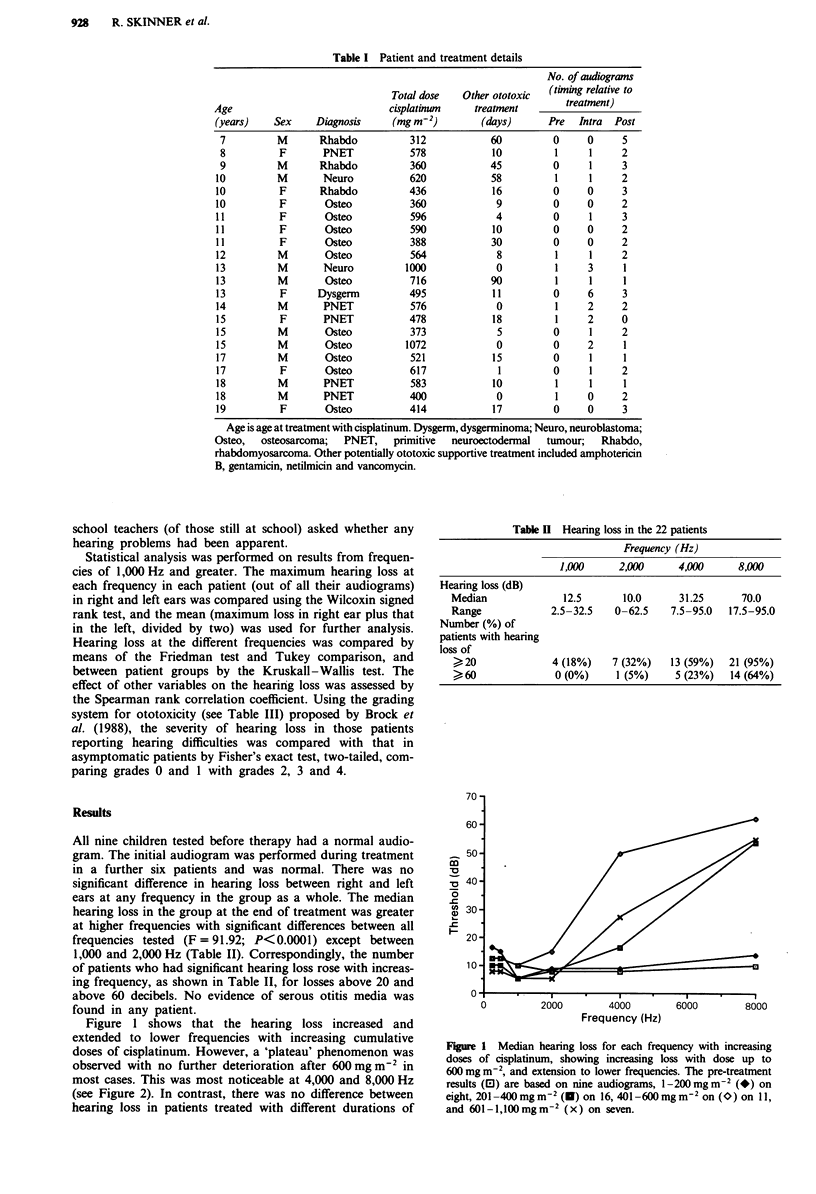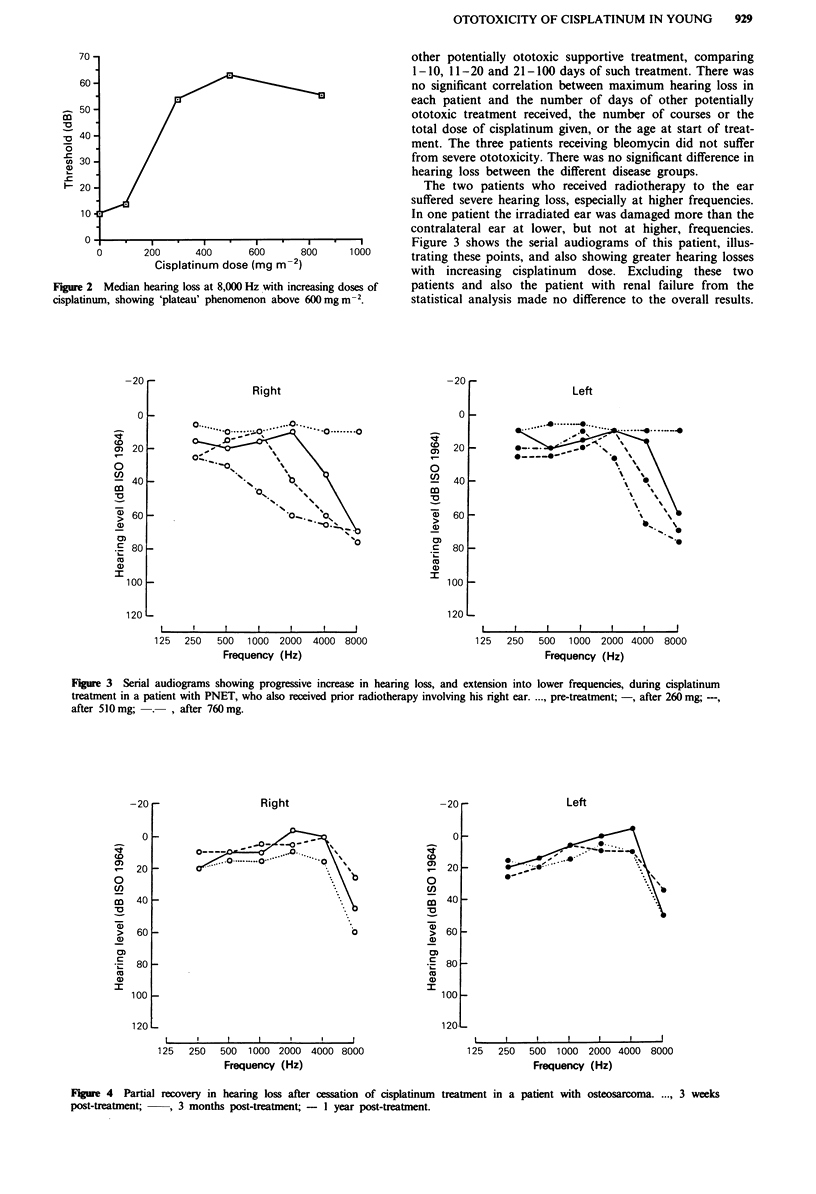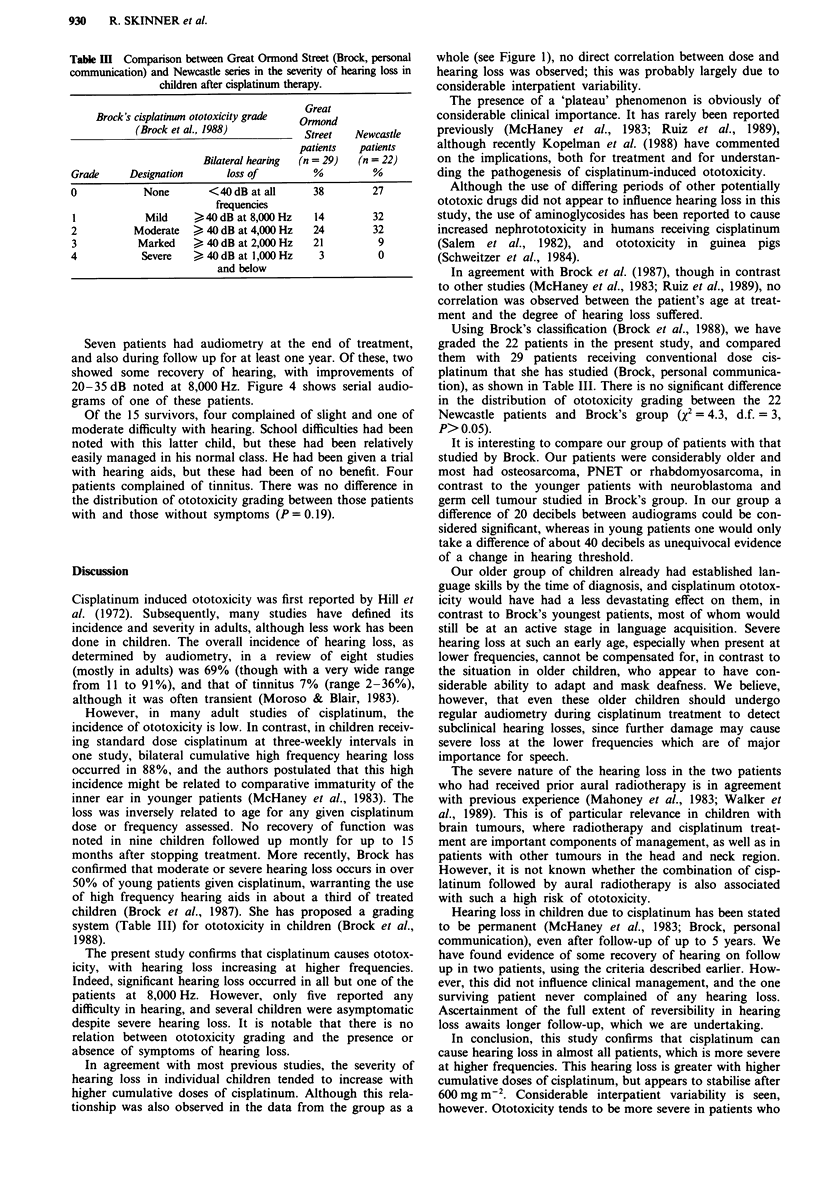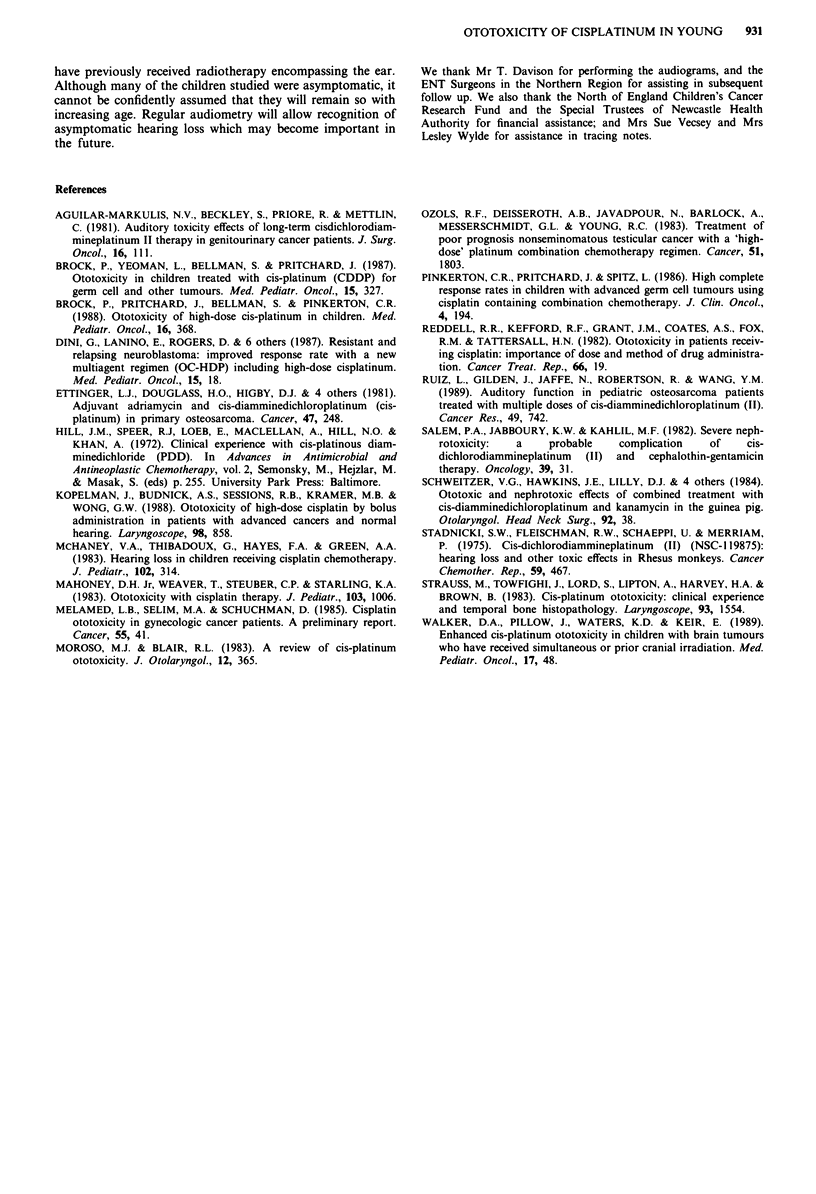# Ototoxicity of cisplatinum in children and adolescents.

**DOI:** 10.1038/bjc.1990.208

**Published:** 1990-06

**Authors:** R. Skinner, A. D. Pearson, H. A. Amineddine, D. B. Mathias, A. W. Craft

**Affiliations:** Department of Child Health, University of Newcastle upon Tyne, Medical School, UK.

## Abstract

Twenty-two children and adolescents who had received cisplatinum for the treatment of solid tumours underwent audiometry to ascertain the extent of hearing damage. Five patients complained of hearing difficulties, causing difficulty at school in one child. Hearing loss greater than 20 decibels occurred in four patients at 1,000 Hz, seven at 2,000 Hz, 13 at 4,000 Hz and 21 at 8,000 Hz. Median hearing loss was greater at higher frequencies (P less than 0.0001), and with increasing cumulative dose of cisplatinum. However, a 'plateau' phenomenon was observed, with no apparent further deterioration in hearing loss at doses greater than 600 mg m-2. Two children who had received prior aural radiotherapy had severe hearing loss. Severe, mostly asymptomatic, ototoxicity is common in children given cisplatinum. However, there is considerable interpatient variability in the hearing loss suffered.


Br. J. Cancer (1990), 61, 927–931 © Macmillan Press Ltd., 1990

### Ototoxicity of cisplatinum in children and adolescents

R. Skinner, A.D.J. Pearson, H.A. Amineddine, D.B. Mathias[1] & A.W. Craft

*Department of Child Health, University of Newcastle upon Tyne, The Medical School, Framlington Place, Newcastle upon Tyne NE2 4HH; and [1]Ear, Nose and Throat Department, Freeman Hospital, Newcastle upon Tyne NE7 7DN, UK.*

**Summary** Twenty-two children and adolescents who had received cisplatinum for the treatment of solid tumours underwent audiometry to ascertain the extent of hearing damage. Five patients complained of hearing difficulties, causing difficulty at school in one child. Hearing loss greater than 20 decibels occurred in four patients at 1,000 Hz, seven at 2,000 Hz, 13 at 4,000 Hz and 21 at 8,000 Hz. Median hearing loss was greater at higher frequencies ($P < 0.0001$), and with increasing cumulative dose of cisplatinum. However, a 'plateau' phenomenon was observed, with no apparent further deterioration in hearing loss at doses greater than 600 mg m$^{-2}$. Two children who had received prior aural radiotherapy had severe hearing loss. Severe, mostly asymptomatic, ototoxicity is common in children given cisplatinum. However, there is considerable inter-patient variability in the hearing loss suffered.

Cisplatinum is a cytotoxic agent which is being increasingly used in the management of paediatric solid tumours. It has contributed to improved response rates in osteosarcoma (Ettinger *et al.*, 1981) and germ cell tumours (Pinkerton *et al.*, 1986). Recent studies have highlighted its efficacy in certain resistant malignancies such as neuroblastoma, and its apparently greater efficacy when used in higher dose regimens (Ozols *et al.*, 1983; Dini *et al.*, 1987). However, its recognised side-effects include nephrotoxicity, ototoxicity, myelosuppression, nausea and vomiting. Renal damage is the major dose-limiting toxicity, but this has been reduced by improved methods of drug administration. Ototoxicity has therefore assumed greater importance, especially in younger children, since it is generally thought to be irreversible, and therefore a potentially serious long term handicap.

Several studies on cisplatinum ototoxicity, predominantly in adults, have yielded different results in relation to its incidence, severity and the occurrence of reversibility (Aguilar-Markulis *et al.*, 1981; Reddell *et al.*, 1982; McHaney *et al.*, 1983; Moroso & Blair, 1983; Brock *et al.*, 1987, 1988; Ruiz *et al.*, 1989). This may be due to variations in the populations studied, different methods of assessing hearing loss, and interactions with other ototoxic treatment.

Cisplatinum-induced damage appears to be mainly confined to auditory function, with few reports of vestibular toxicity, though the latter has seldom been specifically sought. Symptoms of cisplatinum ototoxicity include deafness, tinnitus, and otalgia (Moroso & Blair, 1983). Although it can develop after several courses of treatment, ototoxicity often appears early in treatment (Melamed *et al.*, 1985), and seems to be permanent in most patients (Aguilar-Markulis *et al.*, 1981; Reddell *et al.*, 1982; McHaney *et al.*, 1983; Ruiz *et al.*, 1989). Hearing loss tends to be bilateral, cumulative with further treatment and more severe at higher frequencies, although extension into the speech range can occur (Aguilar-Markulis *et al.*, 1981; Reddell *et al.*, 1982; McHaney *et al.*, 1983; Ruiz *et al.*, 1989). Previous or concurrent use of other ototoxic agents may increase toxicity by more than simple algebraic summation (Aguilar-Markulis *et al.*, 1981). Hearing loss is greater with bolus administration of cisplatinum, rather than infusion (Reddell *et al.*, 1982).

A significant loss of outer hair cells in the lower turns of the organ of Corti in the cochlea of Rhesus monkeys given cisplatinum has been reported (Stadnicki *et al.*, 1975), suggesting a structural basis for ototoxicity, and similar findings have been described in a 9-year-old boy with cisplatinum ototoxicity (Strauss *et al.*, 1983).

The present study aimed to determine the extent of hearing loss in young people treated with cisplatinum, and to identify those patients at greater risk of toxicity.

Correspondence: R. Skinner.
Received 2 May 1989; and in revised form 12 January 1990.

### Patients and methods

Twenty-two patients (12 male) who had received cisplatinum were studied (Table I). Their ages at start of treatment ranged from seven to 19 years, mean 13 years. Eleven had osteogenic sarcoma, five had primitive neuroectodermal tumour (PNET), three had rhabdomyosarcoma, two neuroblastoma and one dysgerminoma. The median total dose of cisplatinum received was 542 mg m$^{-2}$ (range 312–1,072 mg m$^{-2}$) given over two to nine courses (median four). The treatment regimens varied according to the child's diagnosis, with individual cisplatinum dose ranging from 60 to 200 mg m$^{-2}$ per course. The one patient who received 60 mg m$^{-2}$ per course had end stage renal failure and was on continuous ambulatory peritoneal dialysis, and therefore received a low dose protocol. Patients with PNET or neuroblastoma received 200 mg m$^{-2}$ (high dose) cisplatinum in five doses of 40 mg m$^{-2}$ given on consecutive days as a 1 h infusion in hypertonic saline without mannitol but with 3 l m$^{-2}$ intravenous hydration for 7 days. Children with osteogenic sarcoma usually received 100 mg m$^{-2}$ per course as a 24 h infusion with mannitol; those with rhabdomyosarcoma were given 90 mg m$^{-2}$ per course as an 8 h infusion, again with mannitol. All patients received 3 l m$^{-2}$ of intravenous fluids with calcium, magnesium and potassium supplements before, during and after cisplatinum.

Eighteen patients received other potentially ototoxic drugs, including gentamicin, vancomycin, netilmicin and amphotericin B, for a range of from 1 to 90 days (median 10 days). Three patients received one to four courses of bleomycin. Prior radiotherapy fields included both ears to differing degrees in one patient, and one ear in another.

Audiometry was performed in sound proofed rooms by qualified audiology technicians, using either a Kamplex AC4 or a Peters AP6 Clinical Diagnostic Audiometer, calibrated to British Standards (BS2497), TDH39 headphones and MX41AR cushions. The procedure followed was that described by the British Society of Audiology (method A). All patients were examined for evidence of middle ear disease.

A total of 79 audiograms were performed in the 22 patients. The first (baseline) audiogram was performed before any cisplatinum was given in nine patients. Twenty-five audiograms were carried out during the course of treatment. After chemotherapy was completed, 45 audiograms (range 0–5, median 2) were performed during follow-up ranging up to 62 months (median 15).

Significant hearing loss was taken as a deterioration in hearing threshold of 20 decibels or greater at any frequency, while recovery was judged to occur when the threshold improved by 20 decibels or more in at least one frequency in at least one ear in the follow up audiograms.

The 15 surviving patients and their parents were asked if any hearing difficulty or tinnitus had been noted, and the

Table I Patient and treatment details

| Age (years) | Sex | Diagnosis | Total dose cisplatinum ($mg\,m^{-2}$) | Other ototoxic treatment (days) | No. of audiograms (timing relative to treatment) Pre | Intra | Post |
|---|---|---|---|---|---|---|---|
| 7 | M | Rhabdo | 312 | 60 | 0 | 0 | 5 |
| 8 | F | PNET | 578 | 10 | 1 | 1 | 2 |
| 9 | M | Rhabdo | 360 | 45 | 0 | 1 | 3 |
| 10 | M | Neuro | 620 | 58 | 1 | 1 | 2 |
| 10 | F | Rhabdo | 436 | 16 | 0 | 0 | 3 |
| 10 | F | Osteo | 360 | 9 | 0 | 0 | 2 |
| 11 | F | Osteo | 596 | 4 | 0 | 1 | 3 |
| 11 | F | Osteo | 590 | 10 | 0 | 0 | 2 |
| 11 | F | Osteo | 388 | 30 | 0 | 0 | 2 |
| 12 | M | Osteo | 564 | 8 | 1 | 1 | 2 |
| 13 | M | Neuro | 1000 | 0 | 1 | 3 | 1 |
| 13 | M | Osteo | 716 | 90 | 1 | 1 | 1 |
| 13 | F | Dysgerm | 495 | 11 | 0 | 6 | 3 |
| 14 | M | PNET | 576 | 0 | 1 | 2 | 2 |
| 15 | F | PNET | 478 | 18 | 1 | 2 | 0 |
| 15 | M | Osteo | 373 | 5 | 0 | 1 | 2 |
| 15 | M | Osteo | 1072 | 0 | 0 | 2 | 1 |
| 17 | M | Osteo | 521 | 15 | 0 | 1 | 1 |
| 17 | F | Osteo | 617 | 1 | 0 | 1 | 2 |
| 18 | M | PNET | 583 | 10 | 1 | 1 | 1 |
| 18 | M | PNET | 400 | 0 | 1 | 0 | 2 |
| 19 | F | Osteo | 414 | 17 | 0 | 0 | 3 |

Age is age at treatment with cisplatinum. Dysgerm, dysgerminoma; Neuro, neuroblastoma; Osteo, osteosarcoma; PNET, primitive neuroectodermal tumour; Rhabdo, rhabdomyosarcoma. Other potentially ototoxic supportive treatment included amphotericin B, gentamicin, netilmicin and vancomycin.

school teachers (of those still at school) asked whether any hearing problems had been apparent.

Statistical analysis was performed on results from frequencies of 1,000 Hz and greater. The maximum hearing loss at each frequency in each patient (out of all their audiograms) in right and left ears was compared using the Wilcoxin signed rank test, and the mean (maximum loss in right ear plus that in the left, divided by two) was used for further analysis. Hearing loss at the different frequencies was compared by means of the Friedman test and Tukey comparison, and between patient groups by the Kruskall–Wallis test. The effect of other variables on the hearing loss was assessed by the Spearman rank correlation coefficient. Using the grading system for ototoxicity (see Table III) proposed by Brock *et al.* (1988), the severity of hearing loss in those patients reporting hearing difficulties was compared with that in asymptomatic patients by Fisher's exact test, two-tailed, comparing grades 0 and 1 with grades 2, 3 and 4.

### Results

All nine children tested before therapy had a normal audiogram. The initial audiogram was performed during treatment in a further six patients and was normal. There was no significant difference in hearing loss between right and left ears at any frequency in the group as a whole. The median hearing loss in the group at the end of treatment was greater at higher frequencies with significant differences between all frequencies tested ($F = 91.92$; $P < 0.0001$) except between 1,000 and 2,000 Hz (Table II). Correspondingly, the number of patients who had significant hearing loss rose with increasing frequency, as shown in Table II, for losses above 20 and above 60 decibels. No evidence of serous otitis media was found in any patient.

Figure 1 shows that the hearing loss increased and extended to lower frequencies with increasing cumulative doses of cisplatinum. However, a 'plateau' phenomenon was observed with no further deterioration after 600 $mg\,m^{-2}$ in most cases. This was most noticeable at 4,000 and 8,000 Hz (see Figure 2). In contrast, there was no difference between hearing loss in patients treated with different durations of

Table II Hearing loss in the 22 patients

| | Frequency (Hz) 1,000 | 2,000 | 4,000 | 8,000 |
|---|---|---|---|---|
| Hearing loss (dB) | | | | |
| Median | 12.5 | 10.0 | 31.25 | 70.0 |
| Range | 2.5–32.5 | 0–62.5 | 7.5–95.0 | 17.5–95.0 |
| Number (%) of patients with hearing loss of | | | | |
| ⩾20 | 4 (18%) | 7 (32%) | 13 (59%) | 21 (95%) |
| ⩾60 | 0 (0%) | 1 (5%) | 5 (23%) | 14 (64%) |

**Figure 1** Median hearing loss for each frequency with increasing doses of cisplatinum, showing increasing loss with dose up to 600 $mg\,m^{-2}$, and extension to lower frequencies. The pre-treatment results (◻) are based on nine audiograms, 1–200 $mg\,m^{-2}$ (◆) on eight, 201–400 $mg\,m^{-2}$ (■) on 16, 401–600 $mg\,m^{-2}$ on (◇) on 11, and 601–1,100 $mg\,m^{-2}$ (×) on seven.

**Figure 2** Median hearing loss at 8,000 Hz with increasing doses of cisplatinum, showing 'plateau' phenomenon above 600 mg m$^{-2}$.

other potentially ototoxic supportive treatment, comparing 1–10, 11–20 and 21–100 days of such treatment. There was no significant correlation between maximum hearing loss in each patient and the number of days of other potentially ototoxic treatment received, the number of courses or the total dose of cisplatinum given, or the age at start of treatment. The three patients receiving bleomycin did not suffer from severe ototoxicity. There was no significant difference in hearing loss between the different disease groups.

The two patients who received radiotherapy to the ear suffered severe hearing loss, especially at higher frequencies. In one patient the irradiated ear was damaged more than the contralateral ear at lower, but not at higher, frequencies. Figure 3 shows the serial audiograms of this patient, illustrating these points, and also showing greater hearing losses with increasing cisplatinum dose. Excluding these two patients and also the patient with renal failure from the statistical analysis made no difference to the overall results.

**Figure 3** Serial audiograms showing progressive increase in hearing loss, and extension into lower frequencies, during cisplatinum treatment in a patient with PNET, who also received prior radiotherapy involving his right ear. ...., pre-treatment; —, after 260 mg; ---, after 510 mg; —.— , after 760 mg.

**Figure 4** Partial recovery in hearing loss after cessation of cisplatinum treatment in a patient with osteosarcoma. ...., 3 weeks post-treatment; ——, 3 months post-treatment; — 1 year post-treatment.

**Table III** Comparison between Great Ormond Street (Brock, personal communication) and Newcastle series in the severity of hearing loss in children after cisplatinum therapy.

| *Brock's cisplatinum ototoxicity grade (Brock et al., 1988)* | | | *Great Ormond Street patients (n = 29)* | *Newcastle patients (n = 22)* |
|---|---|---|---|---|
| *Grade* | *Designation* | *Bilateral hearing loss of* | *%* | *%* |
| 0 | None | <40 dB at all frequencies | 38 | 27 |
| 1 | Mild | ⩾40 dB at 8,000 Hz | 14 | 32 |
| 2 | Moderate | ⩾ 40 dB at 4,000 Hz | 24 | 32 |
| 3 | Marked | ⩾ 40 dB at 2,000 Hz | 21 | 9 |
| 4 | Severe | ⩾ 40 dB at 1,000 Hz and below | 3 | 0 |

Seven patients had audiometry at the end of treatment, and also during follow up for at least one year. Of these, two showed some recovery of hearing, with improvements of 20–35 dB noted at 8,000 Hz. Figure 4 shows serial audiograms of one of these patients.

Of the 15 survivors, four complained of slight and one of moderate difficulty with hearing. School difficulties had been noted with this latter child, but these had been relatively easily managed in his normal class. He had been given a trial with hearing aids, but these had been of no benefit. Four patients complained of tinnitus. There was no difference in the distribution of ototoxicity grading between those patients with and those without symptoms ($P = 0.19$).

### Discussion

Cisplatinum induced ototoxicity was first reported by Hill *et al.* (1972). Subsequently, many studies have defined its incidence and severity in adults, although less work has been done in children. The overall incidence of hearing loss, as determined by audiometry, in a review of eight studies (mostly in adults) was 69% (though with a very wide range from 11 to 91%), and that of tinnitus 7% (range 2–36%), although it was often transient (Moroso & Blair, 1983).

However, in many adult studies of cisplatinum, the incidence of ototoxicity is low. In contrast, in children receiving standard dose cisplatinum at three-weekly intervals in one study, bilateral cumulative high frequency hearing loss occurred in 88%, and the authors postulated that this high incidence might be related to comparative immaturity of the inner ear in younger patients (McHaney *et al.*, 1983). The loss was inversely related to age for any given cisplatinum dose or frequency assessed. No recovery of function was noted in nine children followed up montly for up to 15 months after stopping treatment. More recently, Brock has confirmed that moderate or severe hearing loss occurs in over 50% of young patients given cisplatinum, warranting the use of high frequency hearing aids in about a third of treated children (Brock *et al.*, 1987). She has proposed a grading system (Table III) for ototoxicity in children (Brock *et al.*, 1988).

The present study confirms that cisplatinum causes ototoxicity, with hearing loss increasing at higher frequencies. Indeed, significant hearing loss occurred in all but one of the patients at 8,000 Hz. However, only five reported any difficulty in hearing, and several children were asymptomatic despite severe hearing loss. It is notable that there is no relation between ototoxicity grading and the presence or absence of symptoms of hearing loss.

In agreement with most previous studies, the severity of hearing loss in individual children tended to increase with higher cumulative doses of cisplatinum. Although this relationship was also observed in the data from the group as a whole (see Figure 1), no direct correlation between dose and hearing loss was observed; this was probably largely due to considerable interpatient variability.

The presence of a 'plateau' phenomenon is obviously of considerable clinical importance. It has rarely been reported previously (McHaney *et al.*, 1983; Ruiz *et al.*, 1989), although recently Kopelman *et al.* (1988) have commented on the implications, both for treatment and for understanding the pathogenesis of cisplatinum-induced ototoxicity.

Although the use of differing periods of other potentially ototoxic drugs did not appear to influence hearing loss in this study, the use of aminoglycosides has been reported to cause increased nephrototoxicity in humans receiving cisplatinum (Salem *et al.*, 1982), and ototoxicity in guinea pigs (Schweitzer *et al.*, 1984).

In agreement with Brock *et al.* (1987), though in contrast to other studies (McHaney *et al.*, 1983; Ruiz *et al.*, 1989), no correlation was observed between the patient's age at treatment and the degree of hearing loss suffered.

Using Brock's classification (Brock *et al.*, 1988), we have graded the 22 patients in the present study, and compared them with 29 patients receiving conventional dose cisplatinum that she has studied (Brock, personal communication), as shown in Table III. There is no significant difference in the distribution of ototoxicity grading between the 22 Newcastle patients and Brock's group ($\chi^2 = 4.3$, d.f. = 3, $P > 0.05$).

It is interesting to compare our group of patients with that studied by Brock. Our patients were considerably older and most had osteosarcoma, PNET or rhabdomyosarcoma, in contrast to the younger patients with neuroblastoma and germ cell tumour studied in Brock's group. In our group a difference of 20 decibels between audiograms could be considered significant, whereas in young patients one would only take a difference of about 40 decibels as unequivocal evidence of a change in hearing threshold.

Our older group of children already had established language skills by the time of diagnosis, and cisplatinum ototoxicity would have had a less devastating effect on them, in contrast to Brock's youngest patients, most of whom would still be at an active stage in language acquisition. Severe hearing loss at such an early age, especially when present at lower frequencies, cannot be compensated for, in contrast to the situation in older children, who appear to have considerable ability to adapt and mask deafness. We believe, however, that even these older children should undergo regular audiometry during cisplatinum treatment to detect subclinical hearing losses, since further damage may cause severe loss at the lower frequencies which are of major importance for speech.

The severe nature of the hearing loss in the two patients who had received prior aural radiotherapy is in agreement with previous experience (Mahoney *et al.*, 1983; Walker *et al.*, 1989). This is of particular relevance in children with brain tumours, where radiotherapy and cisplatinum treatment are important components of management, as well as in patients with other tumours in the head and neck region. However, it is not known whether the combination of cisplatinum followed by aural radiotherapy is also associated with such a high risk of ototoxicity.

Hearing loss in children due to cisplatinum has been stated to be permanent (McHaney *et al.*, 1983; Brock, personal communication), even after follow-up of up to 5 years. We have found evidence of some recovery of hearing on follow up in two patients, using the criteria described earlier. However, this did not influence clinical management, and the one surviving patient never complained of any hearing loss. Ascertainment of the full extent of reversibility in hearing loss awaits longer follow-up, which we are undertaking.

In conclusion, this study confirms that cisplatinum can cause hearing loss in almost all patients, which is more severe at higher frequencies. This hearing loss is greater with higher cumulative doses of cisplatinum, but appears to stabilise after 600 mg m$^{-2}$. Considerable interpatient variability is seen, however. Ototoxicity tends to be more severe in patients who have previously received radiotherapy encompassing the ear. Although many of the children studied were asymptomatic, it cannot be confidently assumed that they will remain so with increasing age. Regular audiometry will allow recognition of asymptomatic hearing loss which may become important in the future.

We thank Mr T. Davison for performing the audiograms, and the ENT Surgeons in the Northern Region for assisting in subsequent follow up. We also thank the North of England Children's Cancer Research Fund and the Special Trustees of Newcastle Health Authority for financial assistance; and Mrs Sue Vecsey and Mrs Lesley Wylde for assistance in tracing notes.

**References**

AGUILAR-MARKULIS, N.V., BECKLEY, S., PRIORE, R. & METTLIN, C. (1981). Auditory toxicity effects of long-term cisdichlorodiammineplatinum II therapy in genitourinary cancer patients. *J. Surg. Oncol.*, **16,** 111.

BROCK, P., YEOMAN, L., BELLMAN, S. & PRITCHARD, J. (1987). Ototoxicity in children treated with cis-platinum (CDDP) for germ cell and other tumours. *Med. Pediatr. Oncol.*, **15,** 327.

BROCK, P., PRITCHARD, J., BELLMAN, S. & PINKERTON, C.R. (1988). Ototoxicity of high-dose cis-platinum in children. *Med. Pediatr. Oncol.*, **16,** 368.

DINI, G., LANINO, E., ROGERS, D. & 6 others (1987). Resistant and relapsing neuroblastoma: improved response rate with a new multiagent regimen (OC-HDP) including high-dose cisplatinum. *Med. Pediatr. Oncol.*, **15,** 18.

ETTINGER, L.J., DOUGLASS, H.O., HIGBY, D.J. & 4 others (1981). Adjuvant adriamycin and cis-diamminedichloroplatinum (cis-platinum) in primary osteosarcoma. *Cancer*, **47,** 248.

HILL, J.M., SPEER, R.J, LOEB, E., MACLELLAN, A., HILL, N.O. & KHAN, A. (1972). Clinical experience with cis-platinous diamminedichloride (PDD). In *Advances in Antimicrobial and Antineoplastic Chemotherapy*, vol. 2, Semonsky, M., Hejzlar, M. & Masak, S. (eds) p. 255. University Park Press: Baltimore.

KOPELMAN, J., BUDNICK, A.S., SESSIONS, R.B., KRAMER, M.B. & WONG, G.W. (1988). Ototoxicity of high-dose cisplatin by bolus administration in patients with advanced cancers and normal hearing. *Laryngoscope*, **98,** 858.

MCHANEY, V.A., THIBADOUX, G., HAYES, F.A. & GREEN, A.A. (1983). Hearing loss in children receiving cisplatin chemotherapy. *J. Pediatr.*, **102,** 314.

MAHONEY, D.H. Jr, WEAVER, T., STEUBER, C.P. & STARLING, K.A. (1983). Ototoxicity with cisplatin therapy. *J. Pediatr.*, **103,** 1006.

MELAMED, L.B., SELIM, M.A. & SCHUCHMAN, D. (1985). Cisplatin ototoxicity in gynecologic cancer patients. A preliminary report. *Cancer*, **55,** 41.

MOROSO, M.J. & BLAIR, R.L. (1983). A review of cis-platinum ototoxicity. *J. Otolaryngol.*, **12,** 365.

OZOLS, R.F., DEISSEROTH, A.B., JAVADPOUR, N., BARLOCK, A., MESSERSCHMIDT, G.L. & YOUNG, R.C. (1983). Treatment of poor prognosis nonseminomatous testicular cancer with a 'high-dose' platinum combination chemotherapy regimen. *Cancer*, **51,** 1803.

PINKERTON, C.R., PRITCHARD, J. & SPITZ, L. (1986). High complete response rates in children with advanced germ cell tumours using cisplatin containing combination chemotherapy. *J. Clin. Oncol.*, **4,** 194.

REDDELL, R.R., KEFFORD, R.F., GRANT, J.M., COATES, A.S., FOX, R.M. & TATTERSALL, H.N. (1982). Ototoxicity in patients receiving cisplatin: importance of dose and method of drug administration. *Cancer Treat. Rep.*, **66,** 19.

RUIZ, L., GILDEN, J., JAFFE, N., ROBERTSON, R. & WANG, Y.M. (1989). Auditory function in pediatric osteosarcoma patients treated with multiple doses of cis-diamminedichloroplatinum (II). *Cancer Res.*, 49, 742.

SALEM, P.A., JABBOURY, K.W. & KAHLIL, M.F. (1982). Severe nephrotoxicity: a probable complication of cis-dichlorodiammineplatinum (II) and cephalothin-gentamicin therapy. *Oncology*, **39,** 31.

SCHWEITZER, V.G., HAWKINS, J.E., LILLY, D.J. & 4 others (1984). Ototoxic and nephrotoxic effects of combined treatment with cis-diamminedichloroplatinum and kanamycin in the guinea pig. *Otolaryngol. Head Neck Surg.*, **92,** 38.

STADNICKI, S.W., FLEISCHMAN, R.W., SCHAEPPI, U. & MERRIAM, P. (1975). Cis-dichlorodiammineplatinum (II) (NSC-119875): hearing loss and other toxic effects in Rhesus monkeys. *Cancer Chemother. Rep.*, **59,** 467.

STRAUSS, M., TOWFIGHI, J., LORD, S., LIPTON, A., HARVEY, H.A. & BROWN, B. (1983). Cis-platinum ototoxicity: clinical experience and temporal bone histopathology. *Laryngoscope*, **93,** 1554.

WALKER, D.A., PILLOW, J., WATERS, K.D. & KEIR, E. (1989). Enhanced cis-platinum ototoxicity in children with brain tumours who have received simultaneous or prior cranial irradiation. *Med. Pediatr. Oncol.*, **17,** 48.